# 266. Cumulative Steroid Dose in Hospitalized Patients and COVID-19 Associated Pulmonary Aspergillosis

**DOI:** 10.1093/ofid/ofac492.344

**Published:** 2022-12-15

**Authors:** Diego Ramonfaur, Jack Salto-Quintana, Nadia Hernández-Mata, Gloria Mayela Aguirre-García, Hiram Villanueva-Lozano, Michel Martínez-Reséndez

**Affiliations:** Harvard Medical School, Baltimore, Maryland; 2Instituto Tecnológico y de Estudios Superiores de Monterrey, School of Medicine and Health Sciences, Monterrey, Nuevo Leon, Mexico; Hospital infantil universitario de Torreón, Torreón, Coahuila de Zaragoza, Mexico; 2Instituto Tecnológico y de Estudios Superiores de Monterrey, School of Medicine and Health Sciences, Monterrey, Nuevo Leon, Mexico; ISSSTE Regional Monterrey, Monterrey, Nuevo Leon, Mexico; Hospital San Jose- Tec Salud, Monterrey, Nuevo Leon, Mexico

## Abstract

**Background:**

Severe COVID-19 elicits a hyperimmune response frequently amenable by high-dose steroids, although treatment may increase the risk for opportunistic infections. Invasive pulmonary aspergillosis (IPA) is a known complication of COVID-19, termed COVID-19 associated pulmonary aspergillosis (CAPA). While steroid use is a known risk factor for CAPA, the role of cumulative steroid dose in the development of CAPA is unclear. This study evaluates the relationship between cumulative steroid dose in hospitalized individuals and the risk for CAPA.

**Methods:**

This retrospective cohort study includes 130 hospitalized patients with RT-PCR-confirmed COVID-19 pneumonia at a specialized center in north Mexico. Patients who developed CAPA were matched by age and gender to two patients who did not develop CAPA. CAPA was defined according to 2020 ECMM/ISHAM criteria. Patients with either possible, probable, or proven CAPA were considered positive cases. Steroid dose was converted to dexamethasone equivalents according to potency and duration. Cumulative dose was obtained in every patient from admission until discharge or diagnosis of CAPA. We assessed the risk of CAPA by the continuous cumulative steroid dose using a logistic regression model.

**Results:**

A total of 42 patients were diagnosed with possible, probable, or confirmed CAPA and were matched to 88 controls. Mean age was 61 ± 14 years, 94 (72%) were male, 11 (12%) were smokers, and 55 (50%) were obese. Mean cumulative steroid dose was 66 ± 75 in patients without CAPA vs 195 ± 226 in patients with CAPA (P< 0.001) (Figure Panel A). The risk for CAPA was higher as the cumulative dose of steroids increased, in a near-linear relationship (OR 1.008; 95% CI 1.003, 1.013, P< 0.001) (Figure Panel B).

**Figure:**
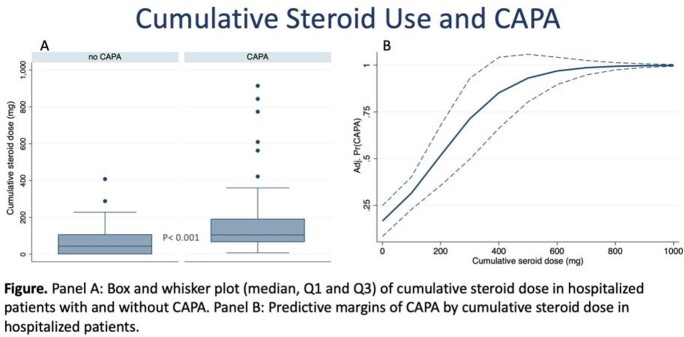

**Conclusion:**

Patients who develop CAPA have a history of higher cumulative steroid dose during hospitalization. The risk for CAPA increases in a near-linear fashion as the cumulative steroid dose during hospitalization increases. While causality cannot be drawn by this study, caution while prescribing high-dose steroids is warranted among individuals hospitalized with COVID-19 pneumonia. Clinical suspicion of CAPA should increase in individuals with a high cumulative dose of steroids and clinical decline.

**Disclosures:**

**All Authors**: No reported disclosures.

